# Supplementary material for: Effect of Artificial Regime Shifts and Biotic Factors on the Intensity of Foraging of Planktivorous Fish
Source: Animals (Basel). 2021 Dec 22;12(1):17. doi: 10.3390/ani12010017 (PMC8749725; doi:10.3390/ani12010017)
Supplement: Supplementary file 1 [file animals-12-00017-s001.zip › animals-1448447 - supplementary material.pdf]

animals

Supplementary material

Effect of Artificial Regime Shifts and Biotic Factors on the Intensity of Foraging of Planktivorous Fish

Krzysztof Ciszewski *, Wawrzyniec Wawrzyniak and Przemysław Czerniejewski

Department of Fisheries Management, Faculty of Food Sciences and Fisheries, West Pomeranian University of Technolgy, Królewicza 4, 71-550 Szczecin, Poland; wwawrzyniak@zut.edu.pl (W.W.); przemyslaw.czerniejewski@zut.edu.pl (P.C.)

* Correspondence: wpfish@wp.pl; Tel.: +48-725-683-011

Citation: Ciszewski, K.; Wawrzyniak, W.; Czerniejewski, P. Effect of Artificial Regime Shifts and Biotic Factors on the Intensity of Foraging of Planktivorous Fish. *Animals* **2022**, *12*, 17. https://doi.org/10.3390/ani12010017

Academic Editor: Constanze Pietsch

Received: 19 October 2021
Accepted: 16 December 2021
Published: 22 December 2021

Publisher's Note: MDPI stays neutral with regard to jurisdictional claims in published maps and institutional affiliations.

Copyright: © 2021 by the authors. Licensee MDPI, Basel, Switzerland. This article is an open access article distributed under the terms and conditions of the Creative Commons Attribution (CC BY) license (https://creativecommons.org/licenses/by/4.0/).

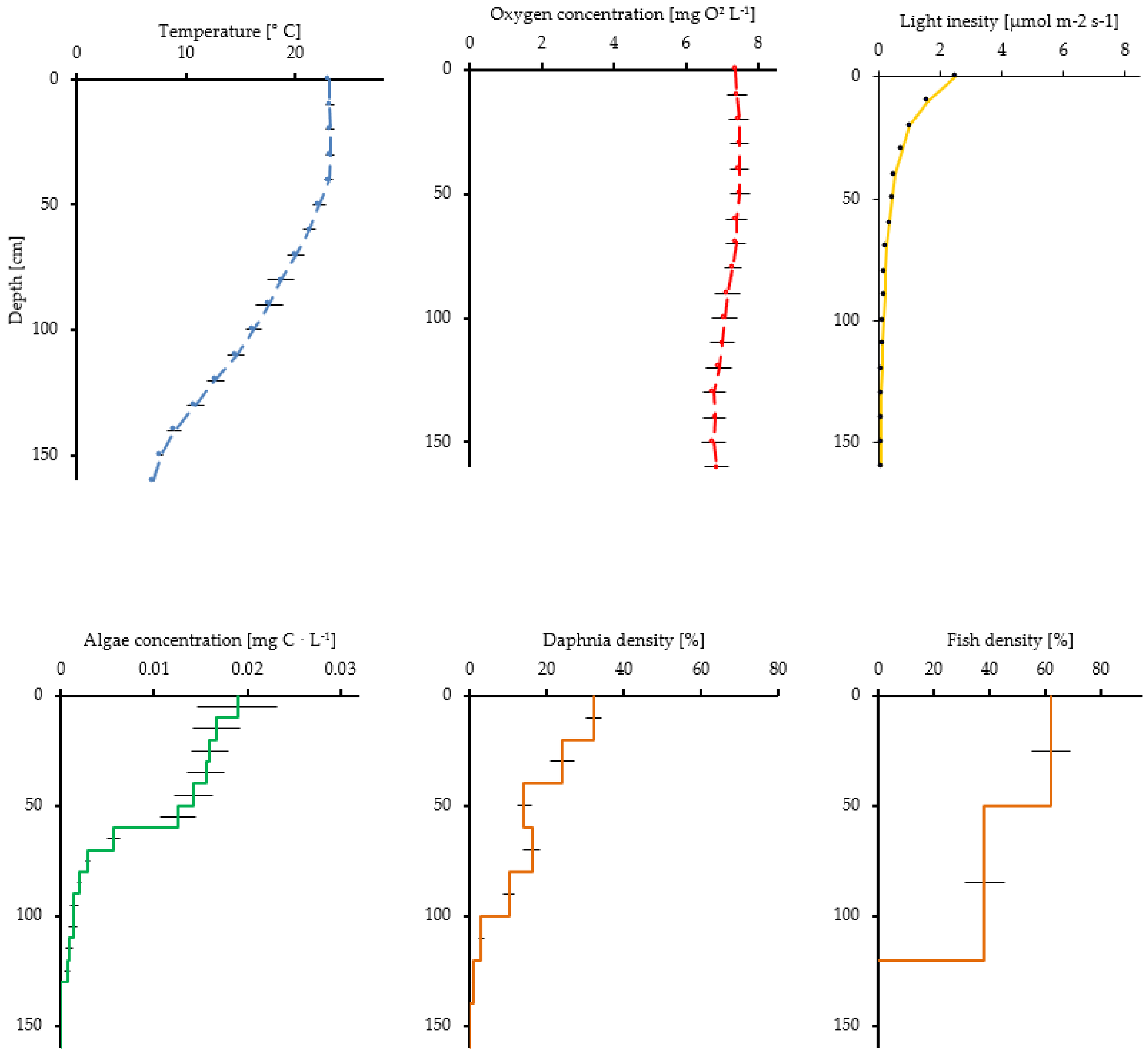

Figure S1. Mild gradient with normoxia LN.

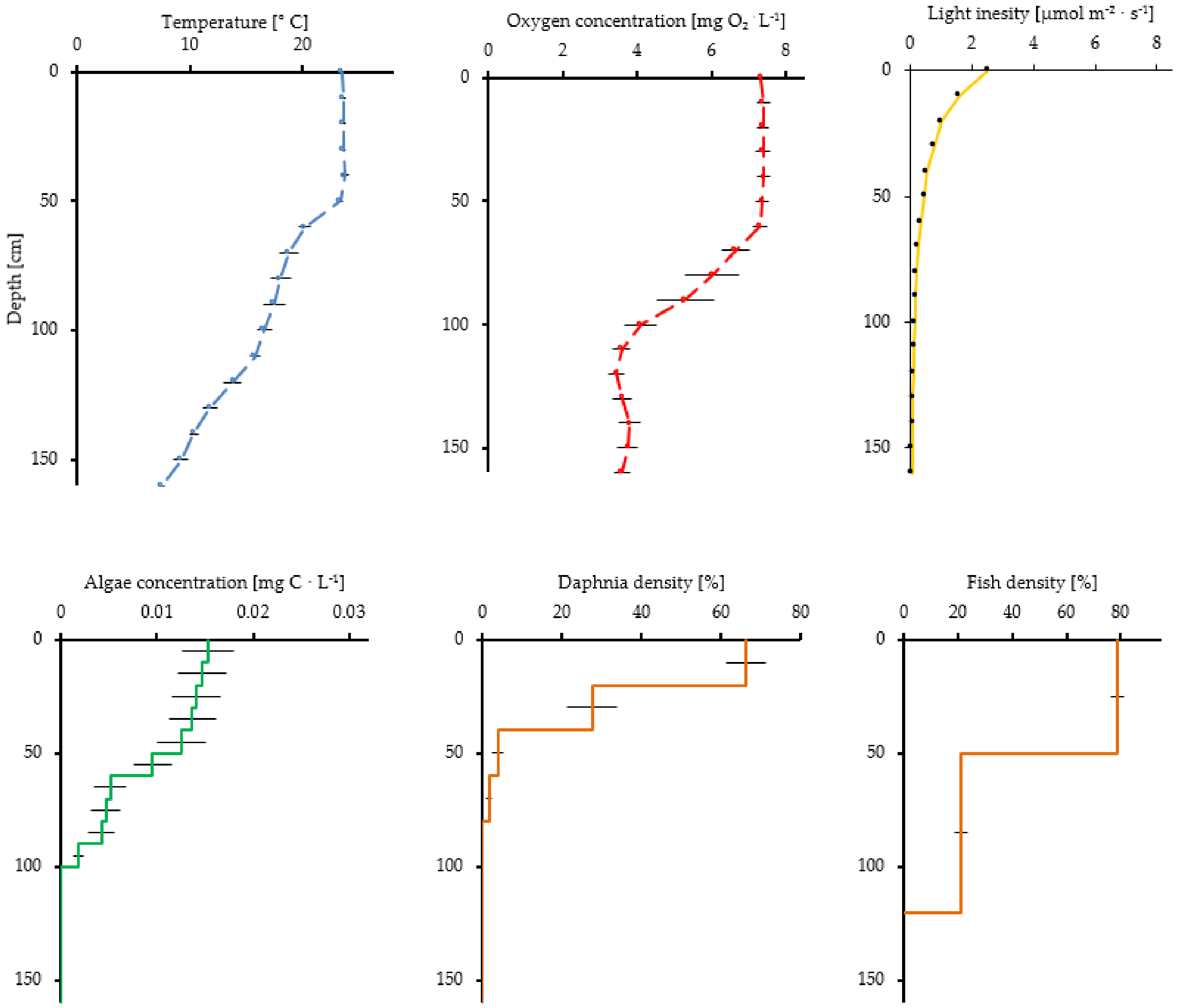

Figure S2. Mild gradient with hypoxia LH, *D.longispina D.pulex*.

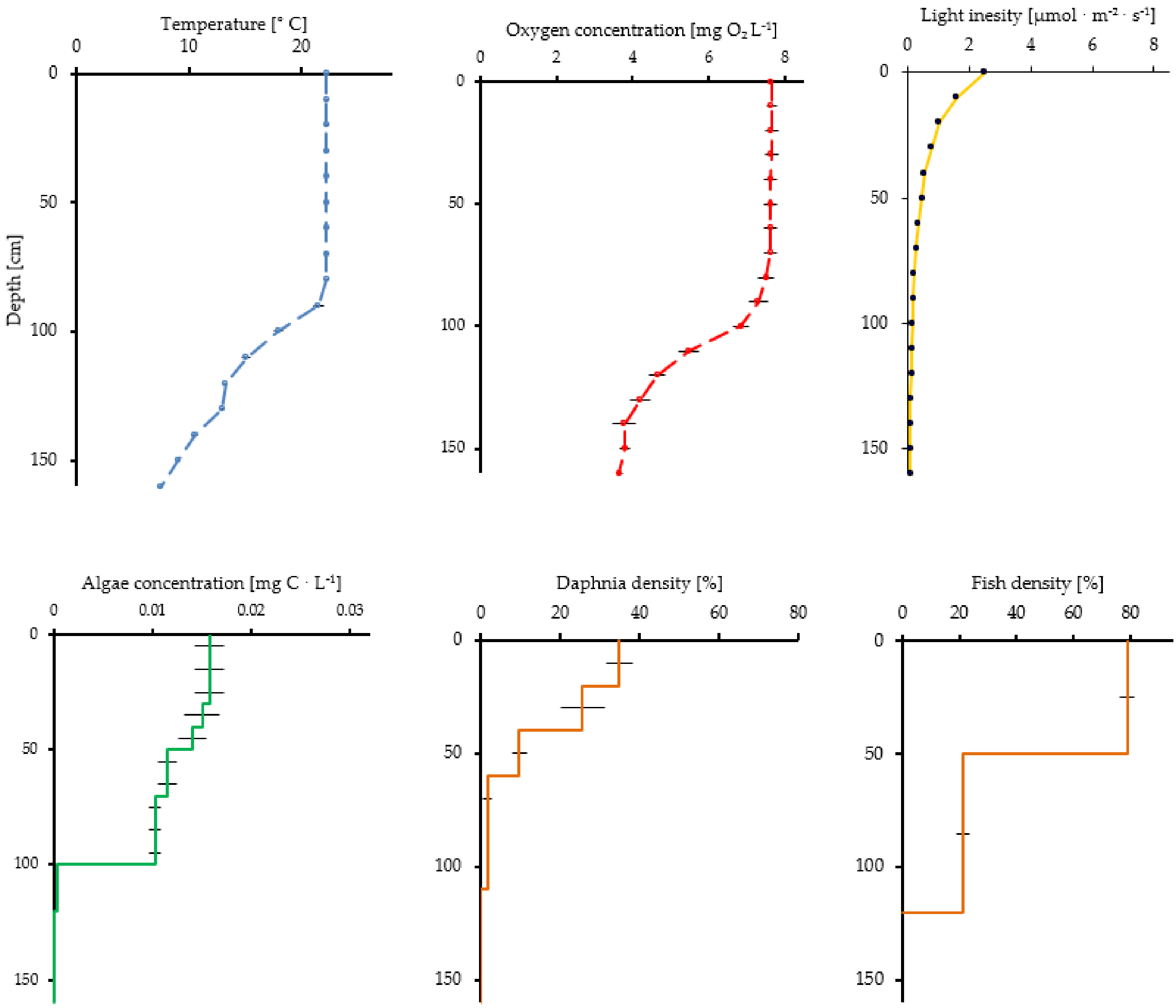

Figure S3. Sharp gradient with hypoxia OH.

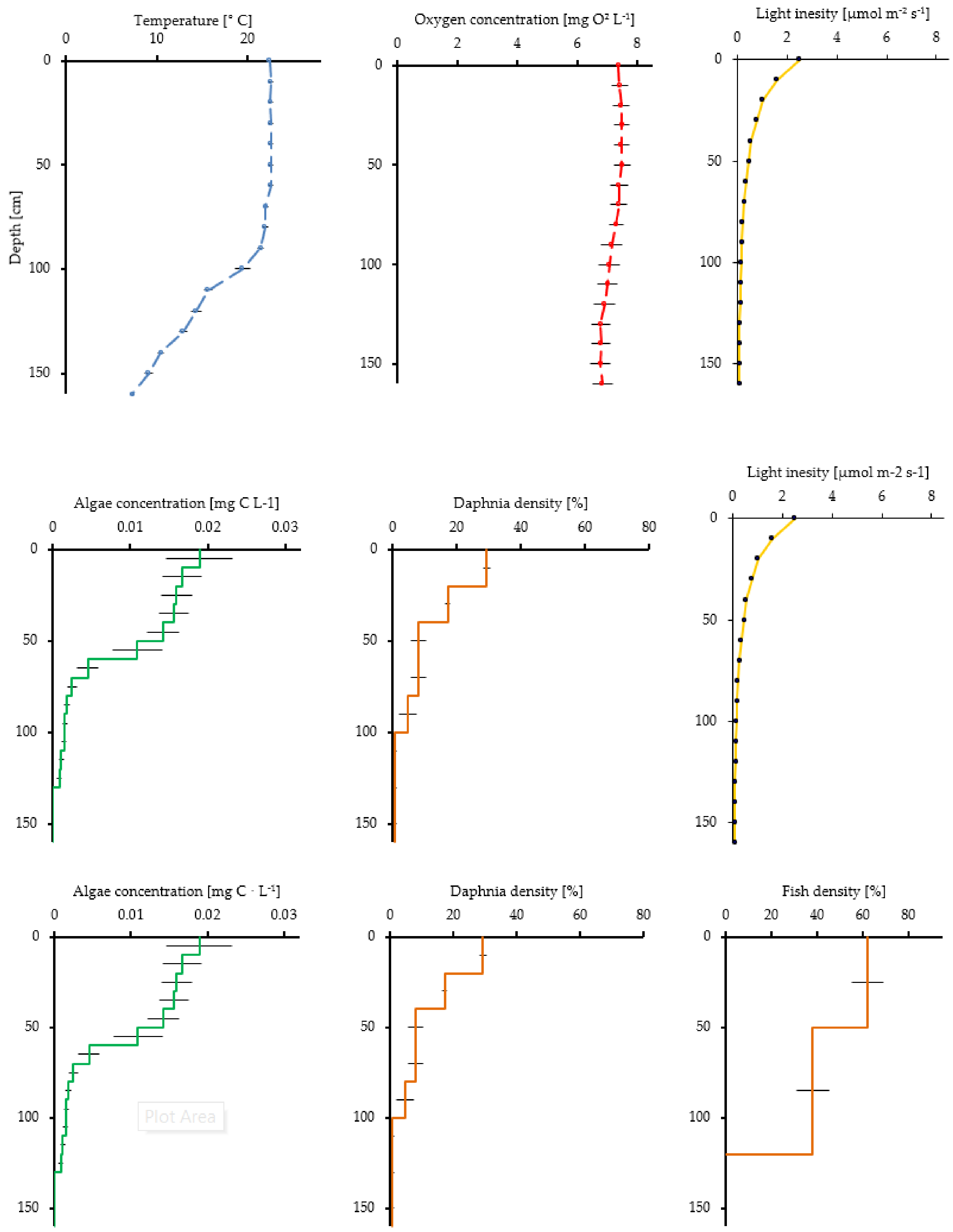

Figure S4. Sharp gradient with normoxia ON4.
